# Facial and Orbital Fractures: A Fifteen Years Retrospective Evaluation of North East Sicily Treated Patients

**DOI:** 10.2174/1874210601711010546

**Published:** 2017-10-31

**Authors:** Michele Runci, Francesco Saverio De Ponte, Roberto Falzea, Ennio Bramanti, Floriana Lauritano, Gabriele Cervino, Fausto Famà, Alessandro Calvo, Salvatore Crimi, Silvia Rapisarda, Marco Cicciù

**Affiliations:** 1 Department of Biomedical, Dental Science and Morphological and Functional Images, Dental School, University of Messina, Messina, Italy; 2 Department of Human Pathology, University of Messina, Messina, Italy

**Keywords:** Midfacial fractures, Orbital fractures, Midface trauma, Hard and soft tissue areas, Road traffic accident (RTA), Craniofacial injuries, Nasoethmoidal

## Abstract

**Background:**

Orbital fractures are classified as diseases usually related to common midface trauma. It represents the most challenging treatment due to the complex anatomy, physiology, and aesthetic role. A midface trauma involves also the zygomatic complex and the nose, however the orbit fracture seems to be a more frequent disease due to its anatomical features.

**Objective:**

The purpose of this work is to retrospectively evaluate and record the frequency of the midfacial traumas and orbital fractures observed in the North Eastern Sicily. The results of the present data may be useful for the clinicians in order to recognize the kind of fracture just from the first general visit having a quick diagnosis and management.

**Methods:**

In the years between 2001 and 2016, about 1200 patients with midfacial trauma and about 100 patients involving the orbital floor have been evaluated. All those patients underwent the surgical fracture reduction and a CT scan follow up control at one month, three months, six months and one year.

**Results:**

Data showed high percentage of orbital floor, nose and mandibular body and ramus fractures; moreover the most frequent causes of fractures seem to be related to motor vehicle accident, followed by assaults, work and fall.

**Conclusion:**

The results have highlighted the changing trends in the causes of facial injuries, particularly the increasing incidence of assaults and the falling incidence of motor vehicle accidents in developed countries. The quick diagnosis and management proved fundamental for the successful treatment. Clinicians should be able to recognize the first symptoms in order to avoid possible complications.

## 1 INTRODUCTION

Maxillofacial fractures usually happen following trauma. Varying from simple, common nasal fractures to gross trauma of the face, management of those diseases can be hardly challenging for surgeons and can negatively prejudice both the psychosocial and functional activities of the patient [1-3]. The midfacial fractures can involve both hard and soft tissue areas of the face, and sometimes, they can be related with other systemic pathologies and requiring multidisciplinary management. However, a protocol for the management of airway, breathing, and circulation is relatively well established [1-4].

The causes related to the maxillofacial fractures are several and their epidemiology can be scheduled on different geographical areas and among different age moments [2, 4]. Road traffic accident (RTA) has been commonly reported as the main etiology of midfacial trauma especially in the developing countries, while assault or fighting seems to be secondary causes. Other minor representative events are sports trauma, domestic accidents, falls or animal bites. Second and third decades of life men are the main involved in the facial fractures cause the frequent engagement in activities that can predispose them to trauma [1-7].

Midfacial trauma management still remains a challenge for the surgeons and clinicians. Permanent functional problems and potentially disfiguring scars can definitely influence the patients ‘quality of life. The skeletal fractures of the middle face are more frequent if compared to mandibular ones. The capability of performing the correct diagnosis and carry out rapid therapies significantly optimizes the functional results with a limitation of aesthetic damages [3-7].

Within the midface fracture, the orbital fractures represent a complex treatment due to the anatomy, and physiology of the ocular district. The orbit principally contributes to the facial appearance and in a midface trauma, it becomes the principal objective of the treatment followed by the zygomatic complex and the nose [1, 2, 7, 8].

Management of orbital fractures is controversial due to the difficulty in evaluating the anatomy of the defected area, and the amount of soft tissue herniation. Orbital fractures represent 40% of craniofacial injuries; the orbital floor is extremely thin and it is frequently damaged among the four walls of the orbit. Accordingly to the literature, these fractures represent up to 84% of cases among the orbital fractures [4-6]. Orbital floor fractures can be classified as pure or impure blowout fractures; the pure fractures exclusively involve the orbital floor while the impure ones are fractures associated with the damage of an orbital rim or other skeletal elements as well as frontal, zygomatic, nasoethmoidal, or maxillary bones [4, 7].

The purpose of this study was to retrospectively analyze the incidence, diagnosis and treatment of midfacial injuries recorded at the University Hospital of Messina (North-East Sicily, Italy) in order to give the clinicians precious information about the diagnosis and management. Moreover, a case of complete orbital trauma management is presented.

## 2 MATERIALS AND METHODOLOGY

The medical data of all the midface trauma patients referred to the Operative Unit of Maxillofacial Surgery, Hospital “Policlinico G. Martino” in Messina, between 2001 and 2016 (15 years) were retrospectively reviewed. Patient’s age, gender, cause of trauma, type of maxillofacial fracture sustained, area of fracture and treatment given were recorded.

Midfacial fractures were also classified accordingly to the involved area in maxillary, zygomatic, orbital, alveolar bone fractures and nasoorbitoethmoidal (NOE) fractures. The patients affected by multiple fractures in the frontal, upper, middle, and lower facial bones, were recorded as panfacial fractures. A total of 1200 patients have been treated. About 100 patients were affected by orbital trauma; 400 patients presented a mandible fracture; about 600 patients needed the management of facial fracture involving maxilla and NOE anatomical districts.

Patients who had refused treatment, had been referred to other facilities out of county or died before treatment, were excluded from study.

The Operative Unit of Maxillofacial Surgery, Hospital “Policlinico G. Martino” in Messina is the only one center in this south area capable of giving first aid and providing management of midface trauma to patients. For this reason, throughout those 15-years, almost all of the maxillofacial injuries in the whole county were treated in our Hospital.

### 2.1 Patients’ Management and Treatment

#### 2.1.1 Mandibular Fracture

The mandibular fractures were quickly identified at the first visit. The loss of patients’ occlusion was the first clinical sign. Then, after x-ray investigation confirming the area of the lesion, mandible fractures required reduction and fixation to allow for primary or secondary bone healing. Condylar fractures have been treated with no surgical approach, but with a functional device.

The mandibular bone healing occurs through a callous intermediate and ensuing ossification. This form of fixation can be obtained with maxilla-mandibular fixation with arch bars, 4-point fixation, occlusal splints, or titanium miniplates.

#### 2.1.2 NOE Fracture

The patients involved in this kind of trauma arrived to the emergency room and operative room for a panfacial traumatic event. NOE treatment objectives are to reinstate the normal appearance of the eyes and nose. Several key parameters, including the intercanthal distance, symmetry and stability of the nasal sidewalls, and nasal projection and contour have to be evaluated before doing the surgery. CT scan is fundamental in order to have quick diagnosis and management. If the patient is to be brought to the operating room for other fractures, one may better assess the need for operative fixation by taking a blunt elevator and placing it intranasally on the medial aspect of the lacrimal fossa. NOE fractures are not easy to treat. Quick diagnosis and surgical management are the keys to have successful outcomes.

#### 2.1.3 Orbital Fracture

**Fig. (1):**
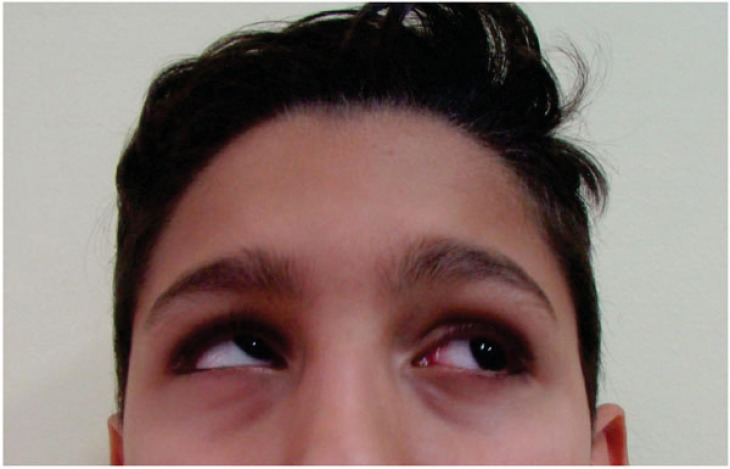
Preoperative frontal view of the patient. The asymmetry of the face and the ocular movements are signs useful for having the suspect of midfacial trauma.

The first visit was performed doing an objective examination including aesthetic evaluation of the patient, highlighting any change in the appearance and facial expressions. Moreover, the pupillary line alignment, possible enophthalmos, the eye movement alterations, the position and movement of the eyelids and the facial asymmetry were recorded. At this stage, the study of the patients face was critical. It was fundamental to perform differential diagnosis between the damage occurred to the elevator palpebrae superior muscle or neural disease referred to the 3^rd^ cranial nerve. Neural problems involve the defect of ocular motility of the four recti muscles. A simple inspection was useful to record the region affected by the trauma, to locate any losses of hard or soft tissue substance and to evaluate the integrity or possible involvement of the optic nerve of the 3^rd^, 4^th^, 6^th^ and 7^th^cranial nerves. According to the literature, a patient in normal health conditions protrudes the eyeballs from the orbital frame by between 18 and 20 mm with a regression of 2 mm. This is the limit beyond which surgical correction is advised. Evaluation of diplopia is fundamental for therapeutic strategy [8] (Fig. **1**).

**Fig. (2):**
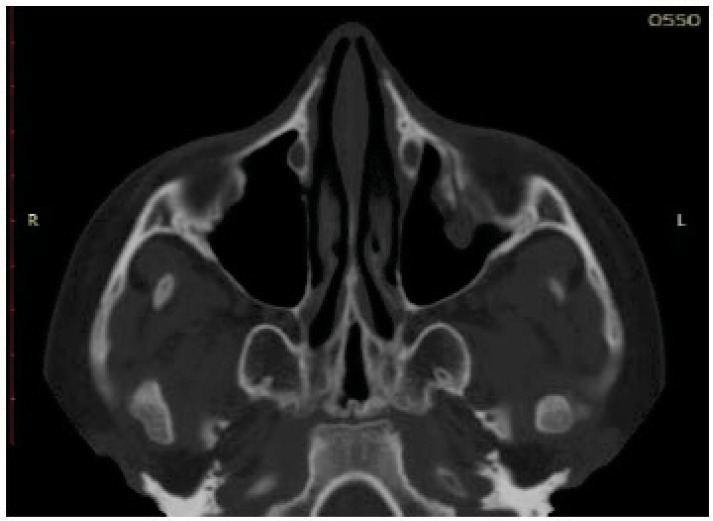
Preoperative CT axial projection. The left side of the sinus showed the presence of soft tissue inside.Preoperative CT axial projection. The left side of the sinus showed the presence of soft tissue inside.

**Fig. (3):**
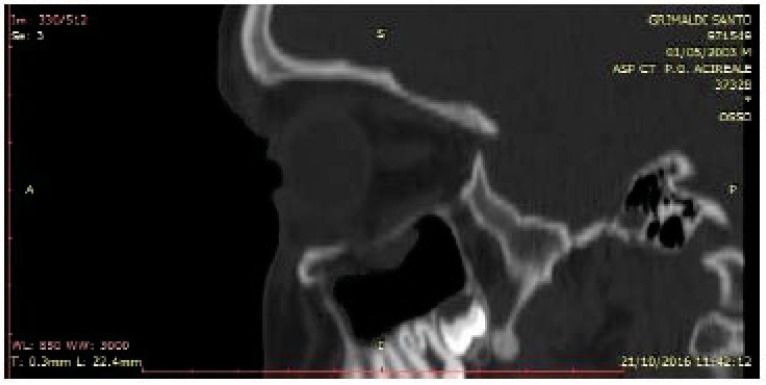
Preoperative CT sagittal ptojection. A particular view of the bone highlight the fracture of the orbital floor.

**Fig. (4):**
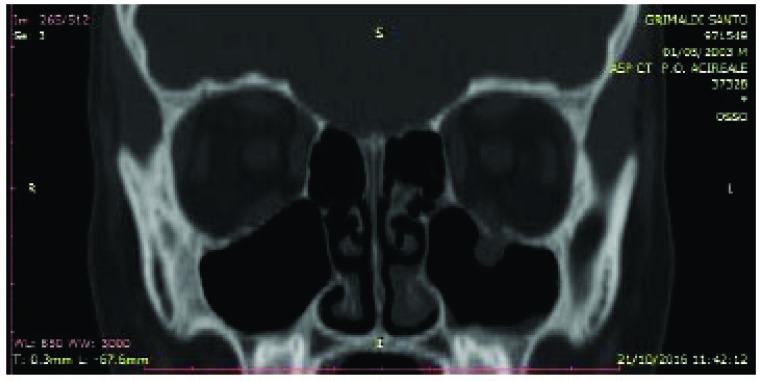
Preoperative CT coronal projection. The left orbital floor seems to be collapsed.

All these patients were subjected to a computed tomography (CT) exam in order to three-dimensionally evaluate the thin orbital layer of the facial projection in the coronal and sagittal sections. This is fundamental for the detection of the orbital floor small size fractures. CT exam provides images of the orbit on the axial, coronal and sagittal planes. The section of the scans should be of maximum thickness of 1 mm; the axial scans should be parallel to the Frankfurt plane on the lateral (the plane passing through the porion and lower orbital point) [8] (Figs. **2**-**4**).

**Fig. (5):**
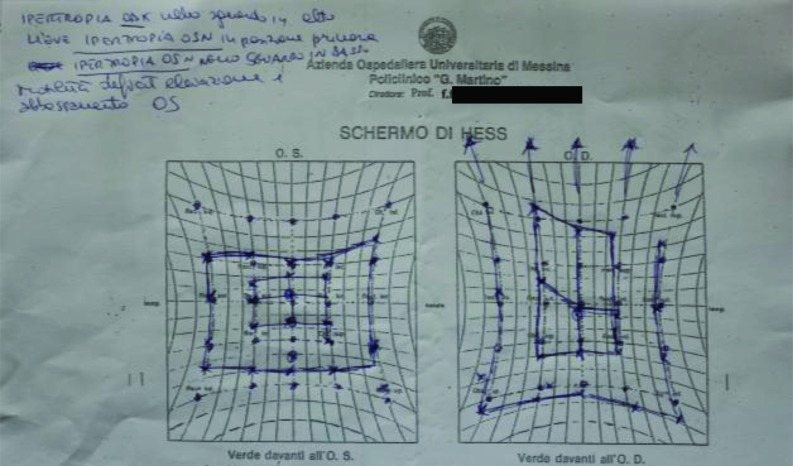
Preoperative screen of Hess-Lancaster.

Furthermore, all patients were requested to perform an eye exam to verify injuries of the globe and fundus. The Hess-Lancaster test was then applied in order to localize the area of ​​the visual field affected by the deficiency and possible diplopia (Fig. **5**).

Once the first examination is completed, the patients are operated under general anaesthesia (GA) after thorough sterilization of the surgical site and the application, if not allergic, of ophthalmic steroid cream to reduce conjunctival damage.

The surgical incision was carried out accordingly with an opening flap 2-3 mm down the lower eyelid and extending from a corresponding point to the top of the lacrimal duct up to 2-3 mm from the corresponding eyelid to. Then, the dissection of the soft tissue was performed by blunt dissection until to localize the lower orbital frame.

Once the fracture of the orbital floor was documented, the techniques used for the restoration of the continuity of the orbital floor can be different depending on the size of the treated defect. In this regard, it is important to remember that high-resolution multi-slides CT preoperatively checked all patients, with coronal and sagittal scan showing the displacement of the floor. In other classification, orbital floor fractures are classified into two types: “small” and “large”. This classification is based upon the measurement of the bony defected area performed on the coronal and sagittal scan of computed tomography [4]. This method consists on limiting two lines corresponding to the fractured floor, calculating the mean value obtained from the higher and the lower line on each view. The elliptical area was calculated by multiplying the width (W on coronal view) and the length (D on sagittal view), then multiplying the result by π. Piombino P [4] classified as “small” the fractures with an area of ​​bony defect less then 3 cm^2^.

**Fig. (6):**
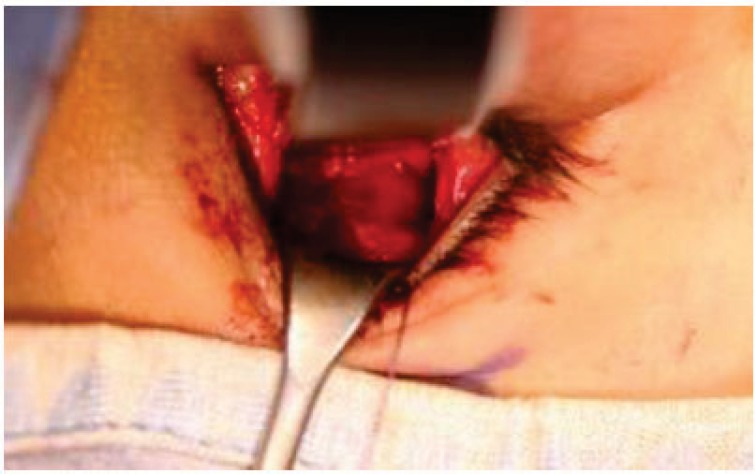
Intraoperative view. Transconjunctival approach. This kind of minimally invasive surgery avoids postepartive discomfort to the treated patient.

**Fig. (7):**
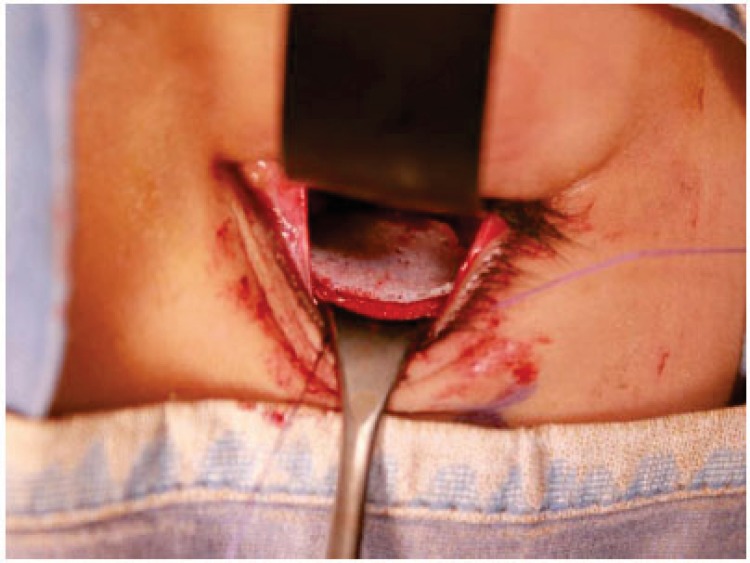
Intraoperative view. Resorbable alloplastic material has been placed in order to re build the orbital floor.

Small orbital fractures were treated with the insertion of a resorbable collagen material; a titanium mesh covered by Medpor was used for the large fractures (Figs. **6**-**7**).

Three days after the surgery, the patients were discharged with administration of antibiotics (Amoxicillin + Clavulanic acid vial 1g iv: 1g x3 / day), anti-inflammatory (Perfalgan vial 1g iv: 1g x3 in the first day, 1g / day from the second day) and with corticosteroids (Soldesam 8mg vial iv: 1 vial x3 / day in the first day, 1 vial x2 / day second day, 1 vial / day on the third day) in order to control the postoperative swelling. The orthotic test was conducted on the third day, after the reduction of most of the swelling, 90% of patients did not report more diplopia in any of the directions.

**Fig. (8):**
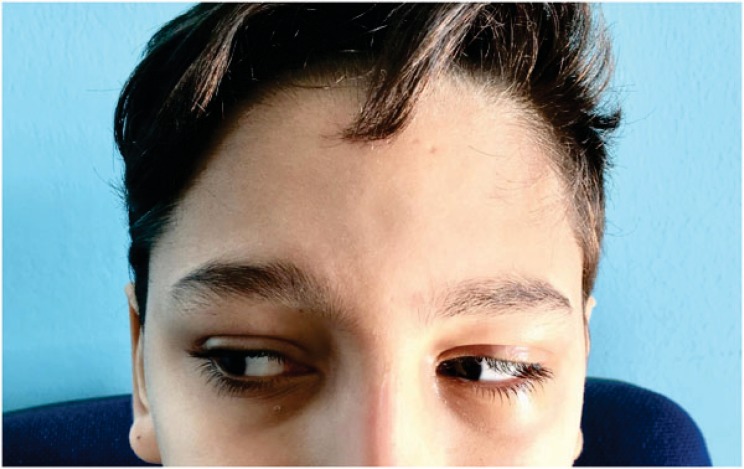
A six month month postoperative control. The young patients had a perfect resolution of the orbital fracture. He referred function and no signs of pain.

A CT scan at one, three, six months and one year post op was performed in all the treated pataitns. At the three months, post-operative investigation of all patients showed the total resorption of the edema. The alloplastic material (collagen) was completely resorbed while the titanium mesh was completely integrated in the healing bone tissue (Fig. **8**).

## 3 RESULTS

A 15-years retrospective evaluation has been performed on 1.200 patients hospitalized and treated at the University Hospital of Messina. The majority of fractures were isolated mandibular fractures (Table **1**; the most frequent cause of fractures seems to be the motor vehicle accident, followed by assaults, work and fall (Table **2**). The mean patients’ age was 35 years, while the average was 37 for men and 33 for women (Table **3**). One patient died during or after treatment and thirty patients experienced different types of ocular and extra-ocular injuries or complications. Other less important complications such as minor post-operative infections or transient motor deficits and rare functional problems such as malocclusion were observed in 5% of fractures (Table **4**).

## 4 DISCUSSION

The traumatic bone fractures localized at the facial area, a common treatment for an Oral and Maxillofacial Surgeon. These fractures happen usually in young people especially males in the third and fourth decades of life because they are majorly involved in many outdoor activities and reckless driving. This assertion is supported by several published studies and is confirmed by the data recorded in our investigation [3, 8-10].

Etiology, type and the area involved in the fractures are related to different factors. However, several investigations reveal how maxillofacial fractures are most commonly caused by trauma such as motor vehicle accidents, alleged assault and falls [1-3, 8-11].

Earlier studies from Europe and the United States of America revealed that traffic accidents are the most frequent causes of facial fractures [12, 13].

The present study carried out in the University Hospital of Messina, confirms that traffic accident is the most frequent cause of maxillofacial fractures. In England, it has been reported that the introduction of the compulsory use of seatbelts has a significant effect on reducing facial injuries [17-20].

Approximately, 70% of maxillofacial fractures occurred in men, the data of the present research confirmed the ratio of 3:1 (“in favour” of the men). However, in many studies, women still commonly sustained fractures as a result of traffic accidents, whilst falls and home assaults were responsible for the remaining fractures in decreasing frequency [19]. In our study, the assaults, accidents at work and sport injuries were the next most common causes.

In Italy, however, road traffic accidents are the leading cause of death up to the age of 29. They were also very common in the next two decades, 30-39 and 40-49.

Road traffic accidents accounted for the largest number of fractures, and the body of the mandible was the most commonly involved site. This observation is also in agreement with the studies by Güven [1], Tanaka *et al.* [9] and Moshy *et al.* [20].

In the recent years, this ratio has become smaller as fractures of the mid-face have increased with more assaults and the increasing road of severe road traffic accidents [8].

This study confirms how the body and the mandible ramus fractures are the most frequent facial injuries treated by maxillofacial surgeons and how the orbital fracture is the most hard to be quickly recognised and managed. This finding confirms the other studies findings where the young people are more involved in midfacial trauma [13-20]. As the index of violence is very well represented by facial injuries, it seems that Italy is a fairly peaceful country as only 10% of such injuries were found to be the result of fights. The most common cause of death in this study was traffic accidents, while the cause of death in 60% was head injuries.

The incidence of naso-frontal fractures is much lower than that of fractures of the mid-face and mandible. Functional consequences follow because of the adjacent structures (eye, lacrimal apparatus, *etc*.) and disruption of the medial canthus or comminution of the frontal sinus and nasal skeleton causes aesthetic problems.

The severe fractures in children under the age of 14 years were due to falls; traffic accidents accounted for a smaller group, and of these, the pedestrian accidents were the most common even if the presence of maxillofacial bone neoplasia seems to be connected with facial skeleton fracture tendency [22-26].

As confirmed by the data of the present work, the quick diagnosis and management of the orbital fracture is fundamental for the final success of the treatment. In the midfacial trauma, the mandibular fractures are easily recognized and managed. The orbital trauma requires more attention to all the clinical signs.

Several papers underlined how urgent ophthalmology evaluation and x-ray CT imaging are recommended in cases of suspected orbital fractures. Earlier intervention is recommended in patients with the evidence of entrapment, in the absence of entrapment or when immediate enophthalmos can be managed conservatively without surgery [21-28].

The decision of the midface fractures management is related to the type of injury, surgeon experience, and available equipment. The orbital trauma is the hardest to be treated for its location and related signs. The surgery can be performed by subciliary, subtarsal, and transconjunctival flaps. The subciliary incision has been associated with a much higher complication rate, with ectropion resulting in approximately 12.9% of cases. The subtarsal one is associated with less ectropion and if placed appropriately, should not result in a conspicuous scar (1% – 3%). Most surgeons prefer the transconjunctival approach to the orbital floor because there is no visible scar and the complication rate is very low, less than 1% in many series. Recently Suzuki *et al.* applied the endoscopic approach to the floor and medial wall. This technique has increased as surgeons try to avoid eyelid complications and improve visualization of the orbital walls; however, the surgery seems to be the more predictable approach for managing the orbital floor fracture. [28, 29].

## CONCLUSION

The major limitation of the present investigation is the retrospective design. However the large number of treated patients can give the clinicians, precious information about midfacial trauma quick diagnosis and management.

Within the limitation of the present study, the recorded data have just highlighted the different etiology events related to the facial injuries. Specifically, the increasing incidence of assaults and the falling incidence of motor vehicle accidents seem to be the main cause of facial trauma in the developed countries.

Orbital fractures require early surgical treatment, and for this reason, a multidisciplinary approach is crucial for having a predictable and successful management.

## Figures and Tables

**Table 1:** The frequency of facial fractures related to the facial bone area.

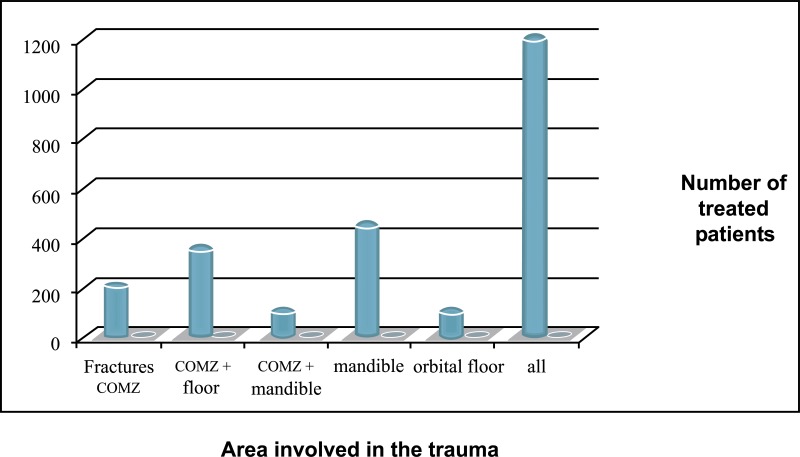

**Table 2:** Frequency of facial fractures classified accordingly the traumatic event.

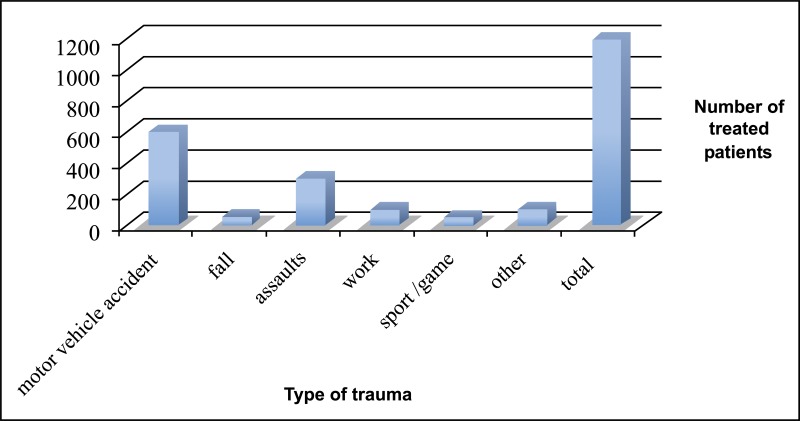

**Table 3:** The mean patients’ age was 35 years, while the average was 37 for men and 33 for women.

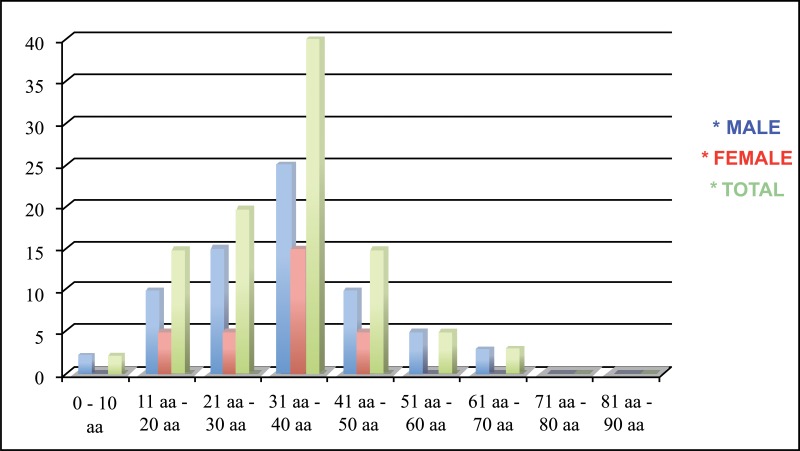

**Table 4:** Associated injures of eye and/or orbital fractures.

Extra-Ocular Lesions Sustained		
Muscles/periorbital soft tissues	Entrapment	6
Position of globe	Enophtalmos	6
	Exophthalmos	1
	Medial displacement	1
Cranial nerve lesions	Oculomotor nerve	2
	Abducens nerve	2
Medial canthal ligament	Telecanthus	14
Lacrimal apparatus	Damage	3
Intra-ocular lesions sustained		
Loss of vision	Optical nerve lesions	5
	Eye ball “destruction”	0
Anterior structures	Corneal laceration	2
	Perforation of gobe	0
Posterior structures	Severe retinal oedema	5
	Macular haematoma	1
	Retinal detachment	1
Total		49
